# Acute Puerperal Uterine Inversion Following a Vaginal Delivery: A Case Report

**DOI:** 10.7759/cureus.110391

**Published:** 2026-06-07

**Authors:** Jaspreet Kaur, Girja S Mohanty

**Affiliations:** 1 Obstetrics and Gynecology, Postgraduate Institute of Medical Education and Research, Chandigarh, IND

**Keywords:** case report, johnson maneuver, obstetric emergency, obstetric labor complications, o negative, postpartum hemorrhage, puerperal uterine inversion, third stage of labor, uterine inversion

## Abstract

Puerperal uterine inversion is a rare and life-threatening obstetric emergency that occurs during the third stage of labor. We report the case of a 27-year-old primigravida who developed an acute third-degree uterine inversion immediately after a vaginal delivery, with partial placental tissue remaining in situ. The patient presented with severe postpartum hemorrhage, hemodynamic shock, and intense pelvic pain. The diagnosis was made clinically, and prompt resuscitative measures (including arrangement of O-negative blood at midnight) followed by uterine reduction via the modified Johnson method were initiated. Early recognition and immediate management are crucial to reduce maternal morbidity and mortality associated with this condition.

## Introduction

Puerperal uterine inversion is a rare, life-threatening obstetric emergency occurring during the third stage of labor, where the uterus collapses inward and turns partially or completely inside out. It is a classic hemorrhagic complication, with an incidence of about one in 2000 to one in 23,000 deliveries [1,2].

Although its exact pathophysiology remains unclear, several predisposing factors have been identified. Extrinsic factors include prolonged labor, excessive or improper traction on the umbilical cord before placenta separation, and inappropriate fundal or abdominal pressure. Intrinsic factors reported in the literature include uterine atony, multiparity, abnormal placental implantation, fundal uterine fibroids, and a short umbilical cord [1-3].

The diagnosis of uterine inversion is primarily clinical. It is classically based on a triad of severe postpartum hemorrhage, hemodynamic shock which is out of proportion to blood loss (as the neurogenic component is also added to the hemorrhagic component), and intense pelvic pain, often accompanied by the absence/indentation of the uterine fundus on abdominal palpation [1,4]. Early recognition is essential, as delayed management significantly increases maternal morbidity and mortality. Management requires immediate resuscitative measures, including the correction of hypovolemia and shock, followed by the prompt reduction of the inverted uterus. Manual reduction (e.g., Johnson's method) or hydrostatic techniques (such as O'Sullivan's method) is the first-line approach; however, when this fails, surgical correction using various techniques like Huntington's procedure or Haultain's incision becomes necessary [3].

We report a successfully treated case of an acute third-degree uterine inversion at the Department of Obstetrics and Gynecology in Postgraduate Institute of Medical Education and Research, Chandigarh, India, highlighting that successful management of uterine inversion relies not only on technical expertise in uterine repositioning but also on strong emergency preparedness, prompt access to rare blood groups, and seamless multidisciplinary collaboration.

## Case presentation

The patient was a 27-year-old primigravida who had delivered in a primary healthcare hospital. A live female neonate was delivered, with an average weight of approximately 2.3 kg. The third stage of labor was complicated by failure of spontaneous placental separation; no forceful cord traction history was elicited. Immediately after delivery, the patient developed profuse vaginal bleeding accompanied by severe pain and the appearance of a vulvar mass, diagnosed as acute uterine inversion. Initial management included a trial of reduction, which failed and after which vaginal packing with two roller gauzes was done, following which she was referred to our institution.

**Figure 1:**
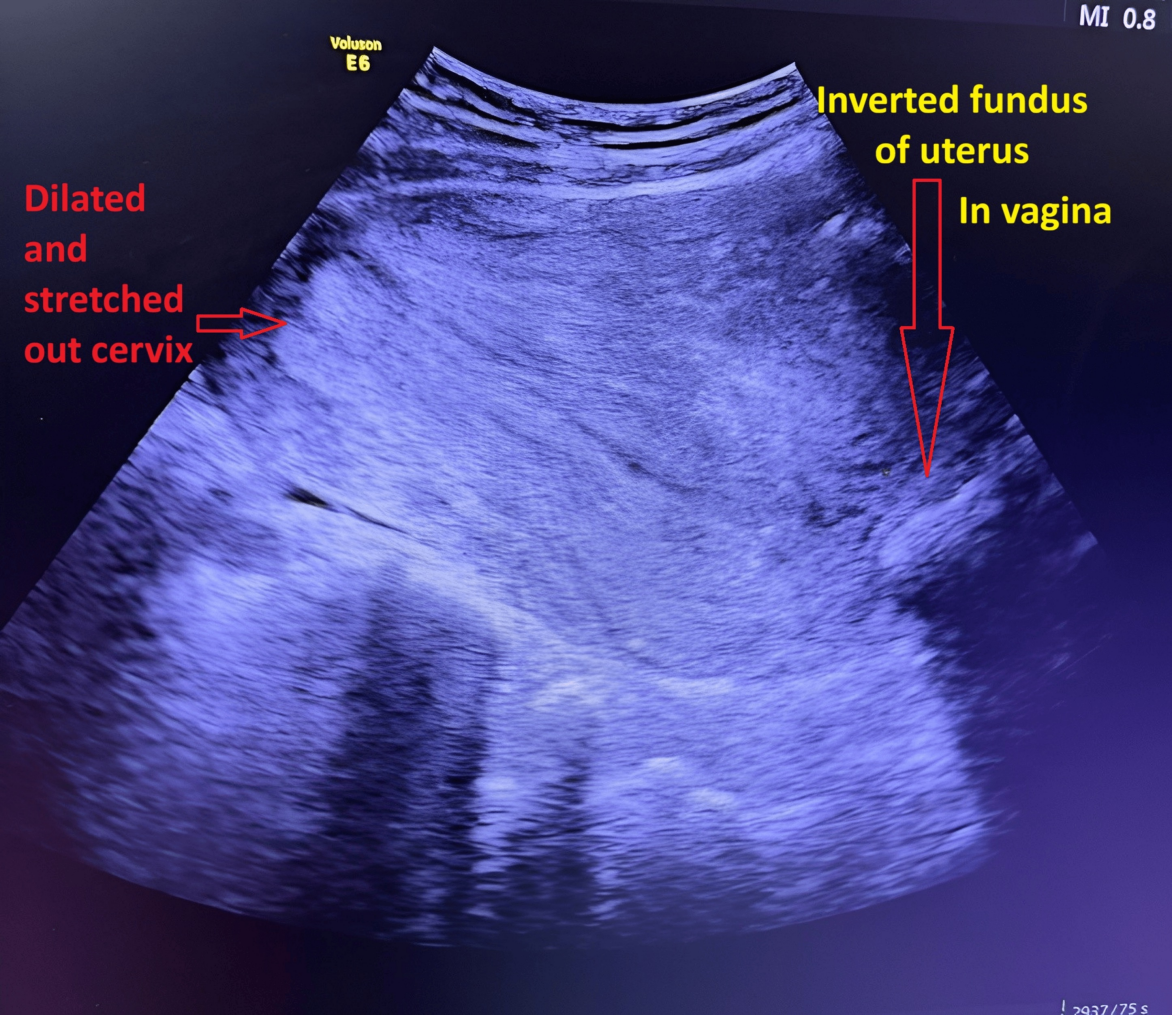
Transabdominal ultrasonography showing the inverted uterus located within the vaginal canal, consistent with a diagnosis of acute puerperal uterine inversion

On presentation to the emergency department, the patient was severely pale, and a blood-soaked pack was inside her vagina. She was in hypovolemic shock, with a blood pressure of 80/60 mmHg, tachycardia (138 beats/min), and tachypnea (26 breaths/min). Clinical examination revealed marked conjunctival pallor and cold extremities. Abdominal examination demonstrated a fundal depression, and local examination showed a completely soaked vaginal pack and perineal pad. Pelvic ultrasonography revealed the fundus was inverted and located within the vaginal canal, confirming the diagnosis of an acute third-degree puerperal uterine inversion (Figure 1). The placenta was out at the primary hospital where she delivered.

Laboratory investigations at admission showed severe anemia with a hemoglobin level of 6.5 g/dL (normal range in females: 12-15.5 g/dL) and leukocytosis (25,000/µL; normal range: 4,000-11,000/µL), while the platelet count was normal, that is, 173,000/µL (normal range: 150,000-450,000/µL).

Immediate management consisted of aggressive fluid resuscitation with crystalloids, and as her blood group was O negative, which was difficult to arrange at midnight, she was managed with fluid initially as a resuscitative measure. In total, four units of crystalloids were transfused as her shock index was 1.7 on admission (a shock index of 0.9-1.1 denotes shock).

The patient was referred to our institution approximately three hours after delivery and was found to have an Rh-negative blood group. In view of her hemodynamic instability and the need for surgical intervention, adequate blood backup was required prior to shifting the patient to the operating theatre. Arranging Rh-negative blood (O negative) during the night resulted in an additional unavoidable delay before we plan for definitive management. In our hospital, admission to intervention duration was less than two hours for such a life-threatening obstetric complication. The patient was taken for uterine repositioning under general anesthesia after preoperative preparation.

Manual reduction was successfully achieved using the modified Johnson maneuver. The non-dominant hand (left hand) was introduced vaginally; the left palm cups the inverted fundus. The left-hand thumb was placed anteriorly for support, and all remaining four fingers were placed posteriorly just at the junction of the inverted uterus. Using the posterior fingers, the most recently inverted portion of the uterus was gradually unfolded in approximately 1 cm increments, with each reduced segment immediately stabilized by the dominant hand placed externally over the fundus. The initial few centimeters of repositioning were technically challenging and were performed cautiously to avoid uterine perforation. Once the posterior uterine wall began to unfold, gentle anteroposterior pressure facilitated smoother and more coordinated reduction, culminating in complete fundal repositioning. Following successful repositioning, the fundus was stabilized by maintaining a clenched fist within the uterine cavity until adequate uterine tone was achieved with oxytocin and per-rectal misoprostol, along with the administration of one vial of intramyometrial carboprost.

After adequate uterine retraction was achieved, the retained placental membranes were completely removed (as the placenta was already out in the primary hospital), with no retained products of conception. The patient stabilized thereafter, and surgical intervention or hysterectomy was not required. Episiotomy repair was performed once hemostasis was secured. Following successful repositioning, oxytocin infusion was given for 12 hours.

The patient received a total of four units of packed red blood cells, two units of fresh frozen plasma, and two units of cryoprecipitate. Her postoperative course was uneventful, and she recovered without complications. She was discharged on the third day of admission in stable condition with a hemoglobin level of 8.5 g/dL on oral antibiotics and analgesics.

**Figure 2:**
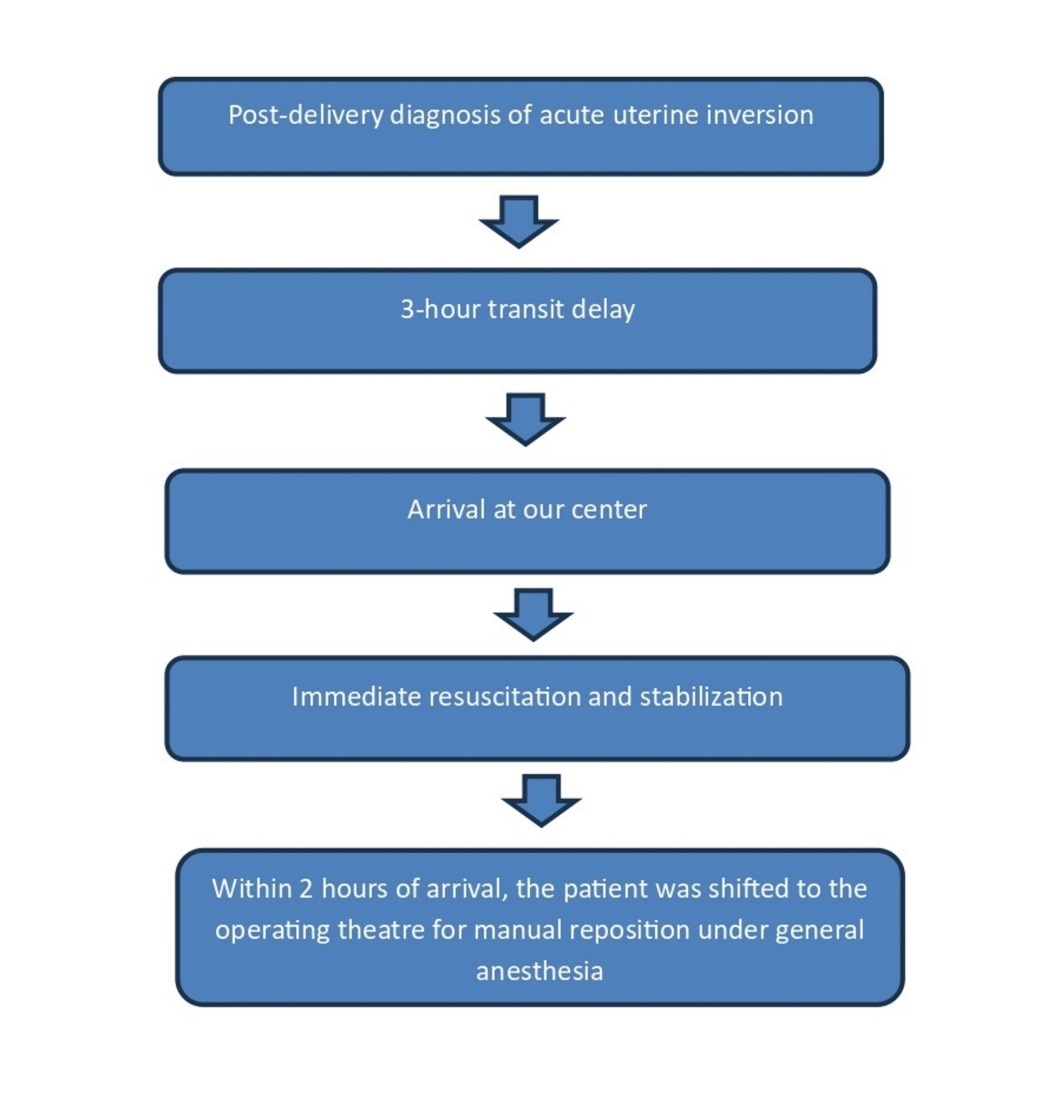
Timeline of events

Figure 2 depicts the timeline of events.

## Discussion

Uterine inversion refers to the inward collapse of the uterine fundus, leading to the partial or complete inversion of the uterus [1]. In incomplete inversion, the fundus remains confined to the uterine cavity, whereas in complete inversion, it extends through the cervix and may, in severe cases, protrude beyond the vaginal introitus [1,4].

The condition is uncommon and can arise in two main contexts: puerperal and non-puerperal. Approximately 95% of cases are puerperal, occurring during or immediately after childbirth, frequently associated with excessive or improper traction on the umbilical cord during the third stage of labor, especially when the placenta is implanted at the fundus [1,2]. Several maternal and obstetric factors may increase susceptibility, including uterine atony, uterine fibroids, and abnormal placental attachment. Additional contributing factors include congenital uterine weakness, excessive fundal pressure, a short umbilical cord, primigravidity, and rapid or precipitous labor. Although some studies have suggested a possible link with the antepartum administration of magnesium sulfate or high doses of oxytocin, this relationship remains unproven [2,3]. Non-puerperal inversion is rare and is usually associated with uterine tumors that lead to the external displacement of the uterus. Uterine inversion is further classified according to the timing of presentation. It is considered acute when it occurs within 24 hours of delivery, subacute when it develops between 24 hours and four weeks postpartum, and chronic if it presents more than one month after childbirth [1].

Uterine inversion may occur either before or after placental separation, and its clinical presentation varies according to the degree of inversion and the timing of onset. In cases of partial inversion, symptoms can be subtle or nonspecific, often making early detection difficult. In contrast, complete inversion usually presents with profuse postpartum hemorrhage, absence of the uterine fundus on abdominal palpation, and signs of maternal cardiovascular compromise [1,4]. Diagnosis is primarily clinical, with bimanual examination revealing the fundus displaced into the lower uterine segment or vaginal canal. When the findings are unclear, ultrasonography can serve as a valuable adjunct to confirm the diagnosis [4].

Early management of uterine inversion is critical to prevent life-threatening consequences. Immediate correction of the inverted uterus is a priority once resuscitative measures have been initiated. The first-line approach is the Johnson maneuver, in which continuous manual pressure is applied to the uterine fundus through the vaginal route to restore normal uterine anatomy [1,3]. However, in the current case, we had modified the Johnson maneuver. The difference is given below in Table 1.

**Table 1:** Difference between the classical Johnson and modified Johnson method (used in this case)

Aspect	Classical Johnson maneuver	Modified Johnson maneuver used in this case
Concept	Single-step manual reposition by sustained upward pressure	Stepwise, controlled unfolding of the inverted uterus
Hand position (vaginal)	The entire hand pushes the fundus upward	The palm cups the fundus; the thumb anterior and four fingers posterior at the inversion junction
Mechanism of reduction	Continuous upward pressure toward the umbilicus	Sequential reduction (~1 cm at a time) of the last inverted segment first
Reduction sequence	Implicit reduction of the posterior wall first	Explicit posterior wall unfolding followed by the anterior wall
External hand role	Usually supportive or optional	Active stabilization of each reduced segment after every step
Force applied	Sustained pressure	Gentle, incremental pressure to minimize perforation risk
Control during initial reduction	May be technically difficult due to the constriction ring	High degree of control during the initial difficult centimeters
Anteroposterior movement	Not specifically described	Deliberate anteroposterior push once the posterior wall begins to unfold
Post-reposition stabilization	Uterotonics after reduction	Intrauterine fist maintained until tone achieved+combined uterotonics (oxytocin, per-rectal misoprostol, intramyometrial carboprost)
Risk mitigation	Rapid reposition prioritized	Emphasis on preventing uterine perforation and re-inversion

This technique is most effective when performed promptly, as a prolonged delay increases blood loss and reduces the success rate. With time, cervical involution results in the formation of a tight constriction ring, making uterine repositioning increasingly difficult. In our case, the delay in the arrangement of O-negative blood group (an unavoidable delay) likely led to the formation of a tight "constriction ring", which is why the initial repositioning of the uterus was "technically challenging". Simultaneously, aggressive fluid resuscitation and hemorrhage control should be done to re-establish maternal hemodynamic stability and prevent fatal outcomes.

As part of management, ongoing oxytocin infusion should be stopped once uterine inversion is diagnosed, and uterine relaxation may be achieved using pharmacological agents. Commonly employed utero-relaxants include terbutaline, salbutamol, and magnesium sulfate. Terbutaline has a rapid onset of action, becoming effective within approximately two minutes, whereas magnesium sulfate typically requires about 10 minutes to produce adequate uterine relaxation. Nitroglycerin, in doses ranging from 50 to 500 µg, has also been reported to be effective in relieving cervical ring constriction [3]. When adequate relaxation cannot be achieved with tocolytic agents, general anesthesia may be considered. Volatile anesthetic agents such as halothane, isoflurane, desflurane, and sevoflurane act as potent uterine relaxants and are particularly useful in hemodynamically unstable patients due to their relatively minimal impact on cardiovascular function [3]. In the present case, general anesthesia with sevoflurane was administered, providing adequate uterine relaxation and allowing successful manual repositioning while maintaining hemodynamic stability. While the modified maneuver was successful, it requires a high level of manual dexterity and may not be feasible in all settings.

Surgical management is indicated when conservative approaches fail to correct uterine inversion. A variety of operative techniques have been described, including the Huntington, Haultain, Spinelli, and laparoscopic methods, with the Huntington and Haultain procedures most commonly employed. Because uterine inversion is a rare condition, sufficiently large cohort studies are unavailable, and definitive comparisons of the success rates of these techniques cannot be made. In rare, life-threatening circumstances, control of severe hemorrhage may necessitate a peripartum or obstetric hysterectomy as a last resort [3,4].

## Conclusions

Uterine inversion, although rare, constitutes a life-threatening obstetric emergency in which maternal outcome is determined by rapid recognition and immediate, coordinated intervention. This case demonstrates that prompt hemodynamic resuscitation combined with early uterine repositioning using a modified Johnson maneuver can be a life-saving and anatomical preservation of reproductive potential, thus acknowledging that while uterine salvage is a success, functional fertility requires long-term follow-up. The stepwise, posterior-first reduction with continuous external stabilization provided greater control during the critical initial phase and minimized the risk of uterine injury and re-inversion.

Equally crucial to the successful outcome was timely access to blood transfusion support. Despite the late-evening presentation at approximately 9:00 pm and the patient's O-negative blood group, rapid arrangement and administration of compatible blood products were made possible through close coordination with the Department of Transfusion Medicine. Manual reduction (via the modified technique) should always be attempted before resorting to surgical incisions like Haultain, even in third-degree inversions. The proactive involvement of the anesthesia team ensured effective resuscitation, hemodynamic stabilization, and safe conduct of the procedure.

This case underscores that optimal outcomes in uterine inversion depend not only on technical expertise in uterine repositioning but also on robust emergency preparedness, availability of rare blood groups, and seamless multidisciplinary collaboration. Adherence to preventive obstetric practices, coupled with swift, well-coordinated emergency management, remains essential for achieving favorable maternal outcomes while preserving future reproductive potential.
